# Parent-Mediated Intervention Training Delivered Remotely for Children With Autism Spectrum Disorder Living Outside of Urban Areas: Systematic Review

**DOI:** 10.2196/jmir.6651

**Published:** 2017-08-14

**Authors:** Dave Parsons, Reinie Cordier, Sharmila Vaz, Hoe C Lee

**Affiliations:** ^1^ School of Occupational Therapy and Social Work Curtin University Perth Australia

**Keywords:** Autistic disorder, Internet, parents, rural health services, telemedicine

## Abstract

**Background:**

Parent training programs for families living outside of urban areas can be used to improve the social behavior and communication skills in children with autism spectrum disorder (ASD). However, no review has been conducted to investigate these programs.

**Objective:**

The aim of this study was to (1) systematically review the existing evidence presented by studies on parent-mediated intervention training, delivered remotely for parents having children with ASD and living outside of urban areas; (2) provide an overview of current parent training interventions used with this population; (3) and provide an overview of the method of delivery of the parent training interventions used with this population.

**Methods:**

Guided by the preferred reporting items for systematic reviews and meta-analyses (PRISMA) statement, we conducted a comprehensive review across 5 electronic databases (CINAHL, Embase, ERIC, PsycINFO, and Pubmed) on July 4, 2016, searching for studies investigating parent-mediated intervention training for families living outside of urban centers who have a child diagnosed with ASD. Two independent researchers reviewed the articles for inclusion, and assessment of methodological quality was based on the Kmet appraisal checklist.

**Results:**

Seven studies met the eligibility criteria, including 2 prepost cohort studies, 3 multiple baseline studies, and 2 randomized controlled trials (RCTs). Interventions included mostly self-guided websites: with and without therapist assistance (n=6), with training videos, written training manuals, and videoconferencing. Post intervention, studies reported significant improvements (*P*<.05) in parent knowledge (n=4), parent intervention fidelity (n=6), and improvements in children’s social behavior and communication skills (n=3). A high risk of bias existed within all of the studies because of a range of factors including small sample sizes, limited use of standardized outcome measures, and a lack of control groups to negate confounding factors.

**Conclusions:**

There is preliminary evidence that parent-mediated intervention training delivered remotely may improve parent knowledge, increase parent intervention fidelity, and improve the social behavior and communication skills for children with ASD. A low number of RCTs, difficulty in defining the locality of the population, and a paucity of standardized measures limit the generalization of the findings to the target population. Future studies should investigate the appropriateness and feasibility of the interventions, include RCTs to control for bias, and utilize standard outcome measures.

## Introduction

Autism spectrum disorders (ASD) are characterized by deficits in social communication and social behavior, including problems interpreting nonverbal gestures, difficulty developing age-appropriate friendships, adherence to rigid routines, and adapting to environmental change [1,2]. In recent years there has been a marked increase in the prevalence of ASD in children with possible reasons cited including (1) advancement in diagnostic procedures, (2) broadening of the diagnosis criteria, (3) increase awareness of ASD, (4) previous diagnosis, and (5) recognition that ASD is a lifelong condition [3]. The current prevalence rate of ASD ranges from 20 per 10,000 to as high as 110 per 10,000 of the global population [3-10].

The increasing prevalence of ASD exerts major demands on early intervention services and education institutions resulting in calls for innovative service delivery models and methods [11,12]. Limited access to adequate health services and a shortage of adequately trained early intervention health and education professionals are of particular concern in regional and remote areas [13-16]. Families of children with ASD who live in regional and remote areas often experience several barriers to improving the outcomes for their child [17], including (1) increased travel distance to suitably qualified clinicians for effective therapy services, (2) delayed diagnosis due to reduced screening programs, and (3) challenges from the inconsistency of health professionals due to high attrition rates and high workforce transition [13,14,16,18,19]. These challenges highlight the need for innovative and alternative early intervention methods for children with ASD and living outside of urban areas.

Effective early intervention requires skilled health and education professionals and places an increased financial and time burden on families to access services [20,21]. As a result, parents or caregivers may be required to play a larger role in the provision of therapeutic services for their children with ASD [22]. To help overcome these barriers, parents can become active agents in the therapeutic process with the appropriate training and ongoing guidance, thereby delivering these interventions to their children in a more consistent manner [23]. This is particularly pertinent for families living outside of urban areas where there is often a lack of access to suitably trained clinicians.

The rise of technological advances in information communication technology (ICT) has paved the way for alternative modes of delivery for health interventions. Evidence suggests that services provided by health professionals using ICT have high efficacy in areas of health, such as delivering behavioral treatment for people with anxiety and depression [24,25]. Moreover, evidence for using telehealth and ICT for children and adolescents with ASD is emerging, with preliminary findings suggesting that it has potential benefits in the diagnosis and delivery of interventions with this population [11,26-32].

Systematic literature reviews support the use of parent-mediated interventions in children with ASD [23,33,34], as does the use of telehealth in providing education sessions to parents or caregivers who have a child with ASD [26,35]. No systematic review has been published on parent-mediated interventions for families having a child with ASD and living outside of urban areas. This is a unique population, and whereas similarities may exist between this group and the general population, these cannot be generalized due to distinctive characteristics and the barriers these families experience due to remoteness [22].

Evidence suggests that the characteristics of families having a child with ASD and living outside of urban areas are unique; however, categorizing and comparing populations across countries is challenging because of differing definitions and classifications systems. For example, in Canada, all territories outside of an urban area are considered to be rural. Rural areas include those “...having a population of at least 1000 and a density of 400 or more people per square kilometer...” [36]. Similarly, according to the US Census Bureau, rural areas include all population, housing, and territory not contained within an urban area [37]. However, the Australian Bureau of Statistics (ABS) uses a 5-category classification based on the Australian standard geographical classification system (ASGC) [38]. The categories include major cities, inner regional, outer regional, remote, and very remote based on a number of variables including population size and distance by road to service centers.

The purpose of this systematic review was to review the existing evidence for parent-mediated intervention training delivered remotely for parents having a child with ASD and living outside of urban areas. In doing so, this review will (1) provide an overview of the studies involving the use of parent-mediated intervention training delivered remotely to parents who have a child with ASD, (2) provide an overview of current parent training programs used with this population, and (3) provide an overview of the method of delivery of parent training interventions used with this population.

## Methods

### Protocol and Registration

The systematic review was registered with PROSPERO (registration number CRD42015027300). The preferred reporting items for systematic reviews and meta-analyses (PRISMA) statement guided the methodology and reporting of this systematic review. The statement provides the structure and transparency considered necessary for reporting systematic reviews in areas of health care.

### Eligibility Criteria

Participants needed to be parents or caregivers of children diagnosed with ASD. With the recent update to the diagnostic and statistical manual of mental disorders (DSM-V), inclusion criteria were expanded to include participants whose children had a diagnosis of autism, Asperger’s syndrome, or pervasive developmental disorder not otherwise specified under criteria of the previous DSM-IV [39]. Studies were included if the children with ASD were aged under 18 years. Given the discrepancies in definition and classification of urban-rural location between countries, for the purpose of this review, we included only those studies in which the population resided outside of major cities or urban areas and authors explicitly described participants as having limited access to services.

Articles were included if the intervention involved training the parents or caregivers in intervention skills to improve the social behavior and communications skills for their child with ASD using telehealth (remote delivery) methods. Face-to-face training, which required parents to travel to a center for training were excluded. Studies were excluded if training was provided solely to therapy professionals or teachers. Telehealth interventions delivered directly and solely by clinicians were excluded from the review, as one study explicitly addressing this issue already exists [26]. Various modes of delivery were accepted for inclusion, including, DVDs, videoconferencing, and Web-based content, as long as the method of delivery enabled remote delivery. Articles of any methodological design that met the eligibility criteria were included, as long as they were published in English in International Scientific Indexing (ISI) listed scientific journals.

### Information Sources

To identify eligible studies, the authors conducted a comprehensive systematic search across 5 electronic databases on July 4, 2016. Databases searched included (1) Cumulative Index to Nursing and Allied Health Literature (CINAHL), (2) Embase, (3) Education Resources Information Center (ERIC), (4) PsycINFO, and (5) Pubmed.

**Table 1:** Search terms.

Database and Search terms	Limitations	Number of abstracts
**Subject Headings:** CINAHL: (MH “autistic disorder”) or (MH “child development disorders, pervasive”) or (MH “pervasive developmental disorder—not otherwise specified”), and (MH “rural health centers”) or (MH “hospitals, rural”) or (MH “rural population”) or (MH “rural health services”) or (MH “rural areas”) or (MH “services for Australian rural and remote allied health”) or (MH “rural health”) or (MH “rural health personnel”) or (MH “telehealth”) or (MH “telemedicine”) or (MH “videoconferencing”) or (MH “teleconferencing”)	English language	80
ERIC: SU.EXACT(“Asperger syndrome”) or SU.EXACT(“pervasive developmental disorders”) or SU.EXACT(“autism”), and SU.EXACT(“rural population”) or SU.EXACT(“rural areas”) or SU.EXACT(“rural youth”) or SU.EXACT(“rural environment”) or SU.EXACT(“rural education”) or SU.EXACT(“teleconferencing”) or SU.EXACT(“telecourses”) or SU.EXACT(“videoconferencing”) or SU.EXACT(“telecommunications”)	English language	29
Embase: autism or Asperger syndrome or “pervasive developmental disorder not otherwise specified,” and (rural health care or rural area or urban rural difference or rural population) or (teleconsultation or telediagnosis or telehealth or telemedicine or telemonitoring or teletherapy or videoconferencing or teleconference or health care delivery)	English language	406
PsycINFO: autism or pervasive developmental disorders or Aspergers syndrome, and (exp rural environments or distance education) or (telemedicine or computer mediated communication or telecommunications media)	English language	64
PubMed **:** (“Autistic disorder” [Mesh] or “child development disorders, pervasive” [Mesh]) and (“rural population” [Mesh] or “rural health services” [Mesh] or “rural health” [Mesh] or “remote consultation” [Mesh] or “telemedicine” [Mesh] or “videoconferencing” [Mesh])	English language	45
CINAHL: Autis* or Asperg* or ASD or (“pervasive,” “developmental,” and “disorder”) or PDD, and (rural* or remote* or regional* or telehealth or tele-health or telemedicine or tele-medicine or telerehab* or tele-rehab* or telediagnos* or tele-diagnos* or teletreat* or tele-treat or teletherap* or tele-therap* or telemonitoring or tele-monitoring or teleintervention or tele-intervention or teletreatment or tele-treatment or telepractice or tele-practice or videoconference* or video-conferenc* or teleconference* or tele-conference* or webbased OR web-based or internet-based or [“technology” and “mediated”] or technology-mediated)	English Language Published date: 20140601-20160704	64
**Free-text search words**		
ERIC:As per CINAHL free text	As per CINAHL free text	45
Embase **:** As per CINAHL free text	As per CINAHL free text	487
PsycINFO **:** As per CINAHL free text	As per CINAHL free text	131
PubMed **:** As per CINAHL free text	As per CINAHL free text	446

### Search Strategy

The categories of search terms used were (1) ASD (autism, autism spectrum disorder, pervasive development disorder not otherwise specified, and Asperger’s’ syndrome) and (2) residing outside of urban areas (rural health, regional health, remote health, telehealth, telemedicine, and videoconferencing) (see Table 1). Search limitations included papers published in English only. A free-text search was completed within the listed databases for literature published from June 16, 2014 to July 4, 2016. The search terms and limitations used for the free-text search are outlined in Table 1. Manual searches of the following journals were performed: The Journal of Rural Health (United States), Australian Journal of Rural Health, Rural Educator, Journal of Research in Rural Education, Australian and International Journal of Rural Education and Rural Special Education Quarterly *.* Finally, manual searches were conducted of the reference lists of articles that met the eligibility criteria.

### Study Selection

The first author screened titles and abstracts of the entire pool of articles that met the inclusion criteria and removed duplicates. Following the removal of the duplicates, all abstracts were screened independently by 2 authors using the inclusion or exclusion criteria. Full-text articles were sourced for abstracts that met inclusion criteria, and articles that did not meet the inclusion or exclusion criteria were excluded. Agreement between authors was reached on 8 out of the 9 included articles. The remaining disagreement was resolved through discussion and consensus.

### Methodological Quality

Methodological quality was assessed using the standard quality assessment criteria as described by Kmet et al [40]. The Kmet appraisal checklist uses a 3-point ordinal system to assess the methodological quality of research papers. The appraisal checklist creates a systematic, quantitative, and reproducible process to assess the methodological quality of a variety of research designs and make comparisons between them. Two authors independently assessed the included articles using the 14-point checklist. Scores were categorized into quality levels: >80% as strong, 70-80% as good, 50-69% adequate, and <50% as limited. The methodological scores are included in Table 2. Disagreements in methodological quality existed between the 2 authors in 2 out of the 9 articles and were resolved through discussion and consensus.

### Data Collection

Data were extracted using comprehensive data extraction forms and grouped under the following headings: (1) aims or objectives, (2) study design, (3) level of evidence, (4) participant characteristics (including geographical location and proximity to services), (5) intervention characteristics, (6) outcome measures, (7) discussion, (8) limitations, and (9) implications for future practice. Data extraction was undertaken by the first author. Data extracted was checked by a second author for accuracy. Only minor discrepancies occurred, and these were resolved through consensus. The level of evidence was determined using the hierarchy of evidence as outlined in the National Health and Medical Research Council (NHMRC) guidelines [41]. Additionally, details of the intervention, dosage, method of delivery, and skills or aims being delivered by the researchers were extracted and summarized. Few studies included in the review had large sample sizes, and the lack of control or comparison groups prevented a meta-analysis.

### Data Items, Risk of Bias, and Synthesis of Results

Participant characteristics were extracted and are represented in Table 3. Kmet ratings were used to assess the risk of bias of at an individual study level [40]. The extrapolated data from this process are represented in Table 2. Characteristics of the extracted data included (1) aims and objectives, (2) study design, (3) level of evidence, (4) intervention characteristics, (5) outcome measures, (6) results, and (7) methodological quality. Significance of data and calculated effect sizes of the interventions were extracted for synthesis. Effect sizes not reported as Cohen *d* were converted for uniformity as appropriate. The magnitude for Cohen *d* effect sizes was interpreted as small≥0.20, medium≥0.50, or large≥0.80 [42]. None of the researchers authored any of the included published studies; hence, no bias in study selection was introduced in conducting the systematic review.

## Results

### Study Selection

**Figure 1:**
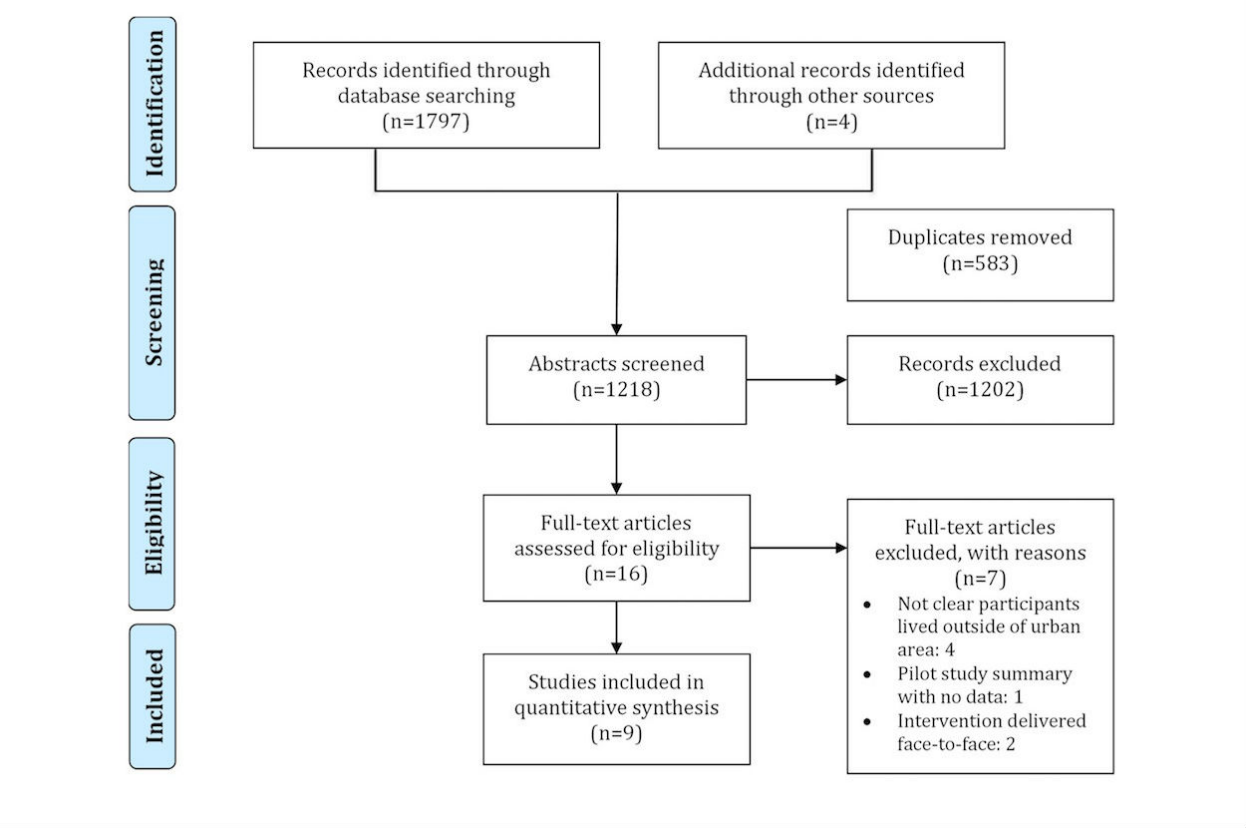
Preferred reporting items for systematic reviews and meta-analyses (PRISMA) flow diagram.

The PRISMA diagram is presented in Figure 1. Database searches yielded 1797 articles. Four additional articles were identified through manual searches of the included studies’ reference lists. From the 2001 articles, 583 duplicates were removed, leaving a total of 1218 abstracts for screening. Following screening of the abstracts, 1202 articles were excluded. The remaining 16 were retrieved for full-text review, and an additional 7 articles were excluded from the study, as participants in four studies were not described as living outside of major cities or urban areas. One study was a summary of a pilot project with no results included, and the 2 remaining studies provided the training to parents in a face-to-face medium. A total of 9 articles met the review eligibility criteria. The articles by Ingersoll and Berger [43], Ingersoll et al [44], and Pickard et al [45] were based on the outcomes from one study, and so they were combined for reporting and discussion throughout this paper.

### Study Design

**Figure 2:**
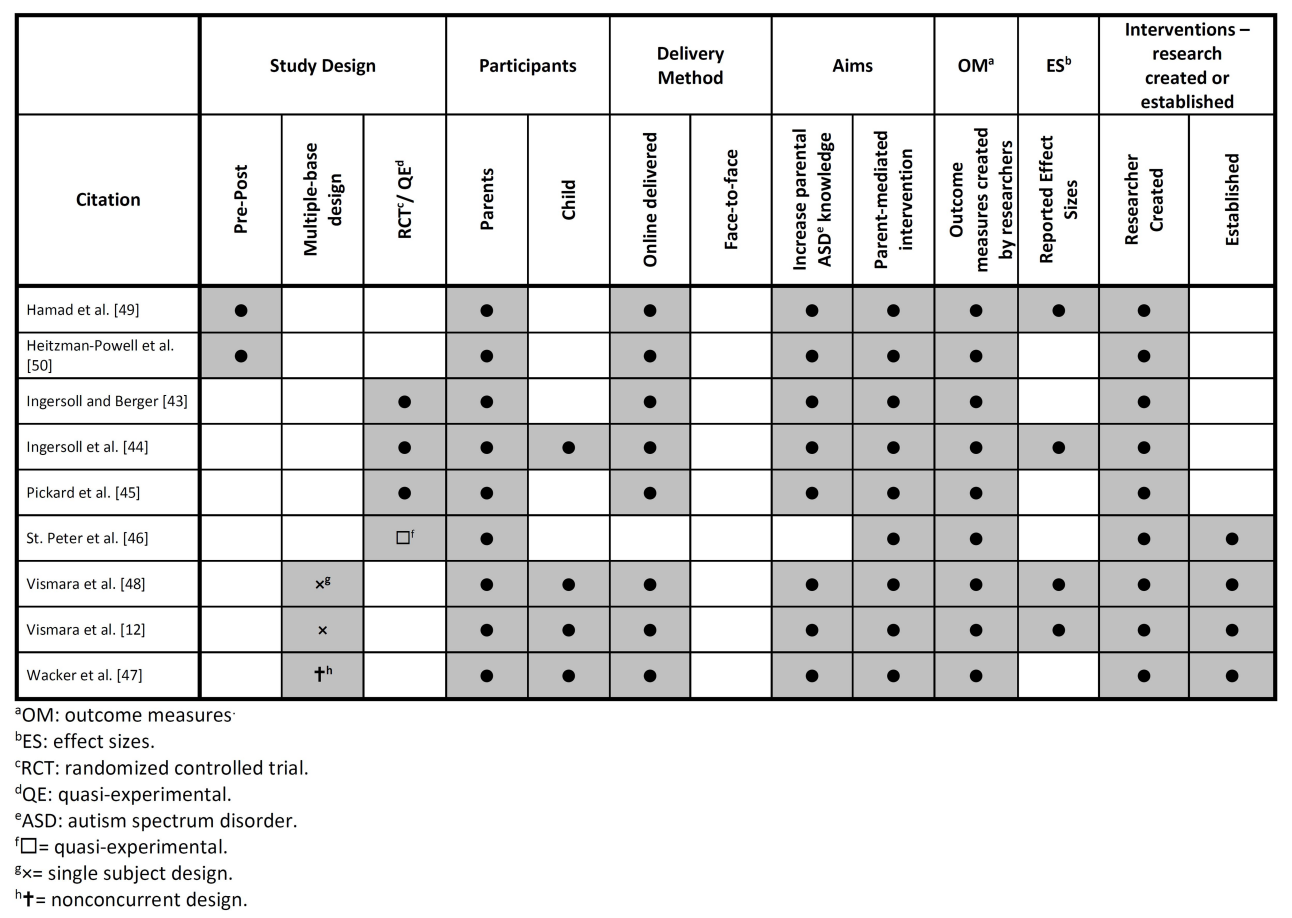
Study Schema.

The 7 studies included 1 quasi-experimental design by St. Peter et al [46], 1 nonconcurrent multiple-baseline designs by Wacker et al [47], 2 single-subject multiple-baseline design by Vismara et al [12,48], 1 RCT each by Ingersoll et al [44] and Pickard et al [45], and 2 prepost test design studies by Hamad et al [49] and Heitzman-Powell et al [50]. A lack of control groups in 5 of the 7 studies precluded the ability to conduct a meta-analysis of the results. An overview of the included papers is provided in Figure 2.

### Level of Evidence

The overall level of the evidence for the studies included in the systematic review was low. The studies by Hamad et al [49], Heitzman-Powell et al [50], Vismara et al [12,48], and Wacker et al [47] demonstrated level IV evidence. The study by St. Peter et al [46] demonstrated level III-1 evidence and the study by Ingersoll et al [44] and Pickard et al [45] was classified as level II [43-45] evidence as per the NHMRC level of evidence guidelines (see Table 2) [41]. The low level of evidence may indicate that it is difficult to conduct research with this population due to travelling distance to research centers and reachability through recruitment strategies, thus making robust study designs more challenging.

**Table 2:** Study characteristics.

Citation and methodology	Aim or objectives	Outcome measures	Results	Methodological quality
Hamad et al [49] Pre- or post-test	Investigate the feasibility of an Internet-based “asynchronous” small-scale three module-Web-based learning course presented in a distance-learning medium.	Parent outcome measures: 25-item Web-based knowledge acquisition measure (test) administered prepost intervention.	Internet-based training curriculum could be effective in training parents about methods and procedures related to behavioral interventions. Pretest scores: mean=68.8, SD=15.6, Posttest scores: mean=82.9, SD=4.9. Large effect size (Cohen *d*=1.21) Paired *t*-tests: mean prepost test scores statistically significant improvement (*P*<.001) for all participants combined (n=51).	Kmet rating: strong (82%) NHMRC^a^level of evidence: level IV
		Child outcome measures: not specified		
Heitzman-Powell et al [50] Pre- or post-test	Evaluate the modified OASIS training intervention for use with parents from a distance.	Parent outcome measures: parent skill assessment in ABA^b^implementation	Implementations of ABA skills (41.23% mean increase)	Kmet rating: good (77%) NHMRC level of evidence: level IV
		Parent knowledge assessment (Web-based) on ASD^c^and ABA principles and procedures	Knowledge assessments (39.15% mean increase)	
		Parent satisfaction with training	High levels of importance and significance of Web-based tutorials (mean scale 1-5:4.62 and 4.71 respectively). High levels of importance and significance of telemedicine coaching sessions (mean scale 1-5:4.62 and 4.8 respectively)	
		Cost savings (driving miles) Child outcome measures	Mean travel savings per family was 2,263 driving miles using telemedicine if compared with face-to-face coaching. Note: Prepost comparison with no statistical analysis for significance.	
		Child outcome measures: not specified		
Ingersoll and Berger [43] Ingersoll et al [44] Pickard et al [45] RCT^d^	Compare parent engagement and effectiveness in self-directed and therapist-assisted versions of a novel telehealth-based parent-mediated intervention for young children with ASD	Parent outcome measures: ImPACT knowledge quiz: 20-item multiple choice quiz taken prepost intervention	Intervention completion was a significant predictor of postintervention knowledge (*P*=.01) in both groups.	Kmet rating: strong (85%) NHMRC level of evidence: level II
		Videotape parent-child interaction for intervention fidelity using the ImPACT intervention fidelity checklist	Intervention completion (*P*=.3) and group assignment (*P*=.45) made significant independent contributions to treatment fidelity. Post intervention fidelity for both groups was significant (*P*=.004) Statistically significant improvement prepost in parent intervention fidelity in both groups (*P*<.01, Large effect size: Cohen *d*=3.21) as well as between groups post intervention (*P*<.01, Large effect size: Cohen *d*=0.3). At follow-up statistically significant (*P*<.001, Large effect size: Cohen *d*=2.92) prepost in both groups but not between groups.	Kmet rating: strong (85%) NHMRC level of evidence: level II
		Parent sense of competence scale	Statistically significant improvement (*P*<.01, Large effect size: Cohen *d*=3.34) prepost intervention in self-efficacy in both groups but not between groups.	
		Parent sense of competence scale	Statistically significant improvement (*P*<.01, Large effect size: Cohen *d*=1.34) prepost intervention in self-efficacy in both groups but not between groups.	
		Family impact questionnaire	Statistically significant improvement (*P*<.05, Large effect size: Cohen *d*=1.03) prepost in parent stress in both groups but not between groups post intervention. Statistically significant improvement (*P*<.05, Large effect size: Cohen *d*=1.47) prepost in positive perception of the child in both groups as well as between groups post intervention (*P*<.05, Large effect size: Cohen *d*=1.16).	
		Parent engagement using website analytics	Therapist-assisted group statistical significantly performed better on parent engagement (number of logins and duration on site) and intervention completion when compared with self-directed groups (*P*<.001 and *P*<.05 respectively)	
		Intervention evaluation survey using 7-point Likert scale measuring treatment appropriateness, website usability, and overall intervention satisfaction.	Participants rated intervention as highly acceptable (mean=6.07, SD=0.79), the website as highly usable (mean=6.36, SD=0.57). Overall satisfaction of intervention was high (mean=6.56, SD=0.71). No statistically significant difference in treatment appropriateness, website usability, and overall intervention satisfaction between groups.	
		49-item 7-point Likert scale quantitative survey administered post intervention examining intervention, appropriateness perceived child social communication gains, burden of the intervention on the family, and frequency of intervention use.	Overall, parent rated intervention favorably with mean scores: 1) Intervention appropriateness 6.59 (SD 0.58), perceived child social communication gains 5.41 (SD 1.24), burden of the intervention on the family 5.72 (SD 1.23), frequency of intervention use 6.36 (SD 0.57) Statistically significant differences between groups (TA^e^vs SD^f^) for intervention appropriateness (*P*=.03, Large effect size: Cohen *d*=0.94) and child social communication gains (*P*=.05, Large effect size: Cohen *d*=0.84). No difference in the burden of intervention on the family and frequency of intervention use domains.	
		Qualitative interviews— semistructured investigated overall perception of intervention and content, perception of feasibility of intervention, experience of support during intervention, and intervention referral preferences.	Qualitative themes: Positive perception of the appropriateness of intervention. The intervention was easy to learn initially but became more challenging as they progressed. The support of a coach would be essential in the later, more complex sections of the intervention. Parents felt more empowered and better able to interact with their child. Perceptions of barriers included time restrictions and technology failure. Parents suggested the intervention should be made available at the time of ASD diagnosis as it may help empower parents at a stressful time.	
		Child outcome measures: language targets	Statistically significant (*P*<.05, Large effect size: Cohen *d*=2.26) prepost improvements in language targets in both groups but not between groups post intervention.	
		MacArthur communicative development inventories: words and gestures	Statistically significant (*P*<.01, Large effect size: Cohen *d*=1.74) prepost improvements in language skills in both groups but not between groups post intervention.	
		Vineland adaptive behavior scales, 2^nd^edition	Statistically significant (*P*<.05, Large effect size: Cohen *d*=1.00) prepost improvements in the communication domain in both groups but not between groups post intervention. No statistically significant differences prepost in the social domains in both groups, however, a statistical difference was observed between groups post intervention (*P*<.05, Large effect size: Cohen *d*=0.91)	
St. Peter et al [46] Quasi-randomized	Compare parental adherence during written or asynchronous video teleconsultation designed to teach parents of children with ASD to implement discrete trial instruction.	Parent outcome measures: Parental adherence between the written (control) and video (experimental) groups Child outcome measures: not specified	Adherence in the video group was significantly higher (*P*<.001) compared with written instructions.	Kmet rating: good (71%) NHMRC level of evidence: level III-1
Vismara et al [48] Single-subject, multiple- baseline design	To assess if a 12-week videoconferencing and DVD learning module (P-ESDM^g^) could improve parents’ acquisition of teaching procedures and result in changes in the child’s social communicative behavior [51].	Parent outcome measures: Eight item, 5-point response scale evaluating parental satisfaction (feasibility and appropriateness) with the support and ease of the intervention	All parents reported satisfaction with support and ease of the telehealth learning intervention. Six parents identified DVD’s as more useful teaching aids compared to handouts. All parents agreed they would recommend an approach to other parents of children with ASD with limited access to community services. Significant increases over time from baseline to follow-up (*P*<.001)	Kmet rating: good (77%) NHMRC level of evidence: level IV
		P-ESDM fidelity tool—5-point Likert rating tool of 13 parent behavior that define the child-centred, responsive interactive style used in PESDM	Significant increases over time from baseline to follow-up (*P*<.001)	
		MBRS^h^—A 5-point Likert rating scale measuring the parent’s style of interacting to or relating to their child.	Significant increases in parental behavior rating from baseline to follow-up in responsivity (*P*<.001), affect (*P*<.001), and achievement orientated behavior (*P*<.001)	
		Child outcome measures: child social communication behavior—10-min videos transcribed and scored for the production of spontaneous and promoted functional verbal utterances and approximations and imitative play actions on objects and gestures.	Significant overall increases from baseline to follow-up in spontaneous functional verbal utterances (*P*<.001), prompted words over time (*P*<.001), and spontaneous imitations (*P*<.001)	
		CBRS^i^[52]—measures engagement and interest in activity as well as joint attention, creativity, and affect demonstrated toward the parent.	Significant increase form baseline to follow-up in child attention (*P*<.001) and child initiation (*P*<.001).	
		MacArthur communicative development inventories: words and gestures	Significant increases from baseline to follow-up with vocabulary production (*P*<.001) and vocabulary comprehension (*P*<.001).	
		Vineland adaptive behavior scales, 2^nd^edition	Significant increase from baseline to follow-up on the adaptive behavior composite (*P*<.05).	
Vismara et al [12] Single-subject, multiple- baseline design	Pilot study of a 12-week telehealth on the Web (videoconferencing and self-guided website) intervention (P-ESDM) and 3-month follow-up to assess: (1) parents’ perception of the intervention as a useful learning platform, (2) parents’ intervention skills and engagement style improvement, (3) website utility to support the intervention, and (4) improvements in the children’s verbal language and joint attention.	Parent outcome measures: Eight item, 5-point response scale evaluating parental satisfaction with the support and ease of the telehealth learning intervention	All parents reported satisfaction with support and ease of the telehealth learning intervention.	Kmet rating: good (77%) NHMRC level of evidence: level IV
		P-ESDM fidelity tool—5-point Likert rating tool of 13 parent behavior that define the child-centred, responsive interactive style used in P-ESDM	Improvement in parent intervention fidelity. Baseline: 0/8 parents meeting criteria for fidelity in tool. Group mean 2.93 (SD 0.6), post intervention: 6/8 parent meeting criteria for fidelity in tool. Group mean 3.69 (SD.51), follow-up: 7/8 parents achieved at least one fidelity score. Group mean 4.15 (SD 0.51)	
		Website use	Average number of logins 30 (SD 18, range 9-60); Average viewing time per day 18 min	
		MBRS [53]—A 5-point Likert rating scale measuring the parent’s style of interacting to or relating to their child.	Improvement in parent engagement style. Baseline: low-moderate with MBRS total score mean=2.91, SD=0.68, post intervention: mean=3.50, SD=0.44, follow-up (3 months): moderate to high range with MBRS total score mean=3.87, SD=0.42	
		Child outcome measures: behavior scoring of videotaped probes—functional verbal utterances and nonverbal joint attention initiations without gestures	Increase in the range of vocalizations at all time points Baseline: mean=2.97, SD=1.93, post interventions: mean=3.60, SD=2.51, follow-up: mean=4.14, SD=2.04 Joint attention initiations remained constant between baseline (mean=1.67, SD=1.07) to post intervention (mean=1.67, SD=1.21) but increased at follow-up (mean=2.16, SD=1.34)	
		MacArthur communicative development inventories: words and gestures	Improvements in VP^j^and comprehension, Baseline: VP mean=111.87, SD=156.03, comprehension mean=224.37, SD=133.25, post intervention: VP mean=163.88, SD=156.03, comprehension mean=284.88, SD=141.53, follow-up: VP mean=213.88, SD=155.08, comprehension mean=314.88, SD= 94.16	
Wacker et al [47] Nonconcurrent multiple baseline design	Conduct functional communication training using coaching from trained behavior analysts to parents via telehealth and compare it with completing the same training in-vivo within families’ homes.	Parent outcome measures: Parent overall appropriateness—7-point Likert scale	Parents rated training as acceptable (mean=6.47. Comparable with in-vivo training (mean=6.18)	Kmet rating: good (73%) NHMRC level of evidence: level IV
		Costs: mileage and consultant costs	Costs through telehealth were considerably lower that for in-home behavior therapy	
		Child outcome measures: Interobserver agreement on child-targeted problem behavior using interval-by-interval comparisons.	Reduction in child-targeted problem behavior when parents coached via telehealth (mean reduction=93.5%). Comparable with in-vivo training (mean reduction=94.1%).	

^a^NHMRC: National Health and Medical Research Council. Designation of levels of evidence: I—Evidence obtained from a systematic review of all relevant randomized controlled trials, II— evidence obtained from at least one properly designed randomized controlled trial, III-1 **—**evidence obtained from well-designed pseudo-randomized controlled trials (alternate allocation or some other method), III-2—evidence obtained from comparative studies with concurrent controls and allocation not randomized (cohort studies), case-control studies, or interrupted times series with a control group, III-3—evidence obtained from comparative studies with historical control, two or more single-arm studies, or interrupted time series without a parallel control group, IV—evidence obtained from case series, either post-test or pre-test and post-test.

^b^ABA: applied behavior analysis.

^c^ASD: autism spectrum disorder.

^d^RCT: randomized controlled trials.

^e^TA: therapist-assisted group.

^f^SD: self-directed group.

^g^P-ESDM: parent model—early start Denver model.

^h^MBRS: maternal behavior rating scale.

^i^CBRS: child behavior rating scale.

^j^VP: vocabulary production.

### Study Participants

For the purposes of this review, study participants were families having a child with ASD, living outside of urban areas, and having limited access to services as reported by the authors. The inherent difficulty of defining regional and remote localities between different countries made delineating study participants based on geography challenging. None of the studies provided quantitative detail about the participants’ proximity and access to services so the interpretation of the findings in relation to this information was impossible. Studies included a total of 197 parents aged between 24 and 69 years involved across the 7 studies. The highest education level achieved by the parents was specified in 5 out of 7 studies. Of the remaining two, one provided a range without specific data and the remaining study did not specify parental level of education. The study populations resided in either the United States, Canada, or Australia. Mothers represented a majority of the parents who received the education and delivered the intervention to the child. Vismara et al [12,48], Wacker et al [47], and the study by Ingersoll et al [44] and Pickard et al [45] measured outcomes for the children receiving the intervention. Refer to Table 3 for detailed demographic information.

### Outcomes

The aim of all of the studies was to improve social behavior and communication skills of children with ASD through increasing the knowledge of parents and caregivers by training them in intervention skills (parent-mediated). Outcome measures varied across all of the studies. All 7 studies used measures created by the researchers. Calculated effect sizes were only possible based on the published information in 3 studies included in the review and are reported in Table 2.

Parental satisfaction and perceptions of appropriateness of the intervention were measured by Vismara et al [12,48], Heitzman-Powell et al [50], and the study by Ingersoll et al [44] and Pickard et al [45]. All reported that parents were satisfied with the training they received. When comparing a therapist-assisted and self-guided website versus a self-guided website only, large effect sizes were recorded in parents’ perception of the appropriateness of the intervention and child social communication gains (Cohen *d*=0.94 and 0.84 respectively) in the study by Ingersoll and Berger [43], Ingersoll et al [44], and Pickard et al [45].

Parents’ self-efficacy was evaluated in the study by Ingersoll et al [44] and Pickard et al [45]. The authors stated that whereas there was a statistically significant (*P*<.01) improvement and a large effect size (Cohen *d*=1.34) preintervention to postintervention for both groups, there was no difference between groups. Parents’ stress levels were not measured prepost interventions in any of the studies.

Knowledge acquisition by parents was measured by Hamad et al [49], Heitzman-Powell et al [50], and in the study by Ingersoll and Berger [43], Ingersoll et al [44], and Pickard et al [45] using quizzes covering the content in the intervention; all studies reported significant increases in knowledge post intervention. Parents’ skills in implementing the acquired therapy techniques were investigated by Heitzman-Powell et al [50], St. Peter et al [46], Vismara et al [12,48], Wacker et al [47], and in the study by Ingersoll and Berger [43], Ingersoll et al [44], and Pickard et al [45]. All of the studies reported statistically significant improvements in parents’ skills in administering skills learnt through the interventions. These findings present evidence that parents who received the appropriate training could gain skills in the delivery of interventions, thus improving the skills in social communication and behavior of their children with ASD.

Vismara et al [12,48] and the study by Ingersoll and Berger [43], Ingersoll et al [44], and Pickard et al [45] utilized the MacArthur communicative developmental inventories [54] to measure the child’s abilities in vocabulary production and comprehension. In all 3 studies, statistically significant improvements were reported in the children’s vocabulary production and comprehension from baseline to follow-up. Again, this provides preliminary evidence that parents who live in geographically isolated areas are able to learn skills in the provision of therapy and implement it appropriately to help improve the communication skills of their children with ASD.

Improvements in social behavior were measured in 2 studies using the Vineland adaptive behavior scales (2nd edition)[55] with Ingersoll and Berger [43], Ingersoll et al [44], and Pickard et al [45] reporting no significant difference prepost intervention and Vismara et al [48] reporting a significant difference in the social domains. Video-recorded interactions of the children with their parents were used in the studies conducted by Vismara et al [12,48] and Wacker et al [47]. All reported statistically significant improvements prepost intervention in joint attention and affect toward the parents with Wacker et al [47] reporting a reduction in child problem behavior.

**Table 3:** Participant characteristics.

Study	No. of participants	Geographical location	Demographics: parent	Demographics: child
Hamad et al [49]	51	“Geographically disparate” in the United States	Gender: male n=4, female n=47 Age: not specified Education level: high school n=6, associate degrees n=0, bachelor degrees n=20, master degrees n=12, other n=3 Note: subgroup demographic breakdown not provided	Gender: not specified Age: not specified
Heitzman-Powell et al [50]	7	Remote areas in the United States	Gender: not specified Age: mean age 37.3 (range=32-47) Education level: ranged from graduate degree to high school diploma. Breakdown not specified.	Gender: not specified Age: not specified
Ingersoll and Berger [43] Ingersoll et al [44] Pickard et al [45]	27	70% (19/27) of participants resided in “rural or medically underserved areas”	Gender: male n=1, female n=26 Age: not specified Education: college degree or higher=16, education levels of remaining participants not specified	Gender: male n=19, female n=8 Age: mean chronological age 3 years, 6 months.
St. Peter et al [46]	32	Rural Appalachian counties in West Virginia, Kentucky, Maryland, Virginia, or Pennsylvania, United States	Gender: male n=11, female n=21 Age: mean age of parents=35.87 years (range, 24-69) Education level: 54.15% had received a college degree. Remaining participant education levels not specified.	Not specified
Vismara et al [48]	8	“Very little access to early intervention services” in California, North Carolina, Arkansas, Texas, and Pennsylvania, United States.	Gender: male n=1, female n=7 Age: not specified Education: not specified	Gender: male n=7, female n=1 Age: mean chronological age 2 years, 4 months (standard deviation=7.6 months, range 16-38 months)
Vismara et al [12]	8	“Minimally available intervention services in their community” in the United States and Canada	Gender: male n=1, female n=7 Age: not specified Education level: college n=4, postcollege n=4	Gender: not specified Age: 1 year n=4, 2 years n=2, 3 years n=1
Wacker et al [47]	17	Regional Iowa, United States	Gender: male n=2, female n=16 Age: mean age 33 years Education level: “most” had some level of postsecondary education. Breakdown not specified.	Gender: male n=16, female n=1 Age: 2 years n=3, 3 years n=4, 4 years n=3, 5 years n=5, 6 years n=2 (range 29-80 months)

**Table 4:** Intervention characteristics.

Study	Intervention description and dosage	Method of delivery to parent	Skills or aims of intervention
Hamad et al [49]	Web-based training intervention in behavioral interventions Dosage: intervention approximately 4-8 h within a 3-week period Three modules	On the Web using Blackboard Vista 4 platform Included: short Web-based lectures, practical exercises, video demonstrations of procedures, study questions, and frequent short Web-based quizzes.	• Positive reinforcement: selection and use of reinforcement. • Relationship building: parent and teaching cooperation. • Prompting and prompt fading.
Heitzman-Powell et al [50]	OASIS training intervention Research-to-practice Applied behavior analysis outreach training model Dosage: Eight modules; timeframe not specified	Training program combines Web-based instructional modules and participation in distance coaching sessions.	• Introduction to ASD^a^and behavioral treatment; • Basic ABA^b^principles and procedures • Use ABA procedures to teach new skills • Use ABA procedure to reduce challenging behavior • Generalize skills to other settings • Collection and analysis of data for data-based intervention decision-making • Working with treatment teams and other provider
Ingersoll and Berger [43] Ingersoll et al [44] Pickard et al [45]	Project ImPACT on the Web—Website-based training for a naturalistic, developmental-behavioral, parent-meditated intervention for children with ASD Dosage: Self-directed—Encourage to complete 1 lesson per week, approximately 80 min for 12 weeks. Therapists assisted—dosage same as self-directed group plus 2 30-min remote coaching sessions per week by trained therapist.	Access to training material was on the Web via personal computer. Included: narrated slideshow with embedded video examples of techniques, written description of lessons, exercises, homework, and reflection questions Training program for the therapist-assisted group was administered by trained therapists using videoconferencing software.	• Promote child social communication within the context of play and daily routines
St. Peter et al [46]	Implementation discrete-trial instructions using a video training materials Dosage: video training was 37 min in duration Written training was a 30-page manual	Written training materials (control) or video training materials (experimental) containing similar content.	• Increase adherence to discrete-trial instruction procedures.
Vismara et al [48]	Parent early start Denver model (P-EDSM) training Dosage: Once-per-week, 1 h parent training sessions for 12 weeks	Telehealth delivery using live, 2-way conferencing with a qualified therapist and the provision of a DVD including all intervention materials with the addition of video recorded examples of the therapist demonstrating skills.	• Increasing child’s attention and motivation • Using sensory social routines • Promoting dyadic engagements and joint activity routines • Enhancing nonverbal communication • Building imitation skills • Facilitating joint attention • Promoting sequence relations • Employing promoting, shaping, and fading techniques • Conducting functional assessments of behavior to develop new interventions.
Vismara et al [12]	Parent early start Denver model (P-EDSM) training Dosage: Once-per-week, 1.5 h parent training sessions for 12 weeks	Telehealth delivery using live, 2-way conferencing with a qualified therapist and a self-guided website.	• Increasing child’s attention and motivation • Using sensory social routines • Promoting dyadic engagements and joint activity routines • Enhancing nonverbal communication • Building imitation skills • Facilitating joint attention • Promoting sequence relations • Employing promoting, shaping, and fading techniques • Conducting functional assessments of behavior to develop new interventions.
Wacker et al [47]	Functional communication Dosage: Weekly 60 min sessions until completion of treatment,	Telehealth using PC and video-monitors from behavior consultants	• Child taught to comply with task request and then to mand for a break to play • Child requesting toys after having to wait for increasing period of time • Request attention when adult attention was removed.

^a^ASD: autism spectrum disorder^.^

^b^ABA: applied behavior analysis.

In summary, it appears that interventions targeting parents’ knowledge and including fidelity checks have statistically significant improvements with large effect sizes when reported. Additionally, large to small effect sizes were reported in the child’s improvement in social behavior and communication skills when reported within the studies.

### Interventions

All interventions were developed with consideration of the geographical isolation of participants, with the aim to ease administration of the intervention and increase feasibility of delivery. Parent training interventions investigated in the included articles are summarized in Table 4.

All interventions were developed by the researchers and varied in dosage and method of delivery. This variation makes synthesis of the research challenging and limits the generalizability of these methods of intervention to the targeted population. Dosage for the interventions ranged from an intensive format of 5 h per day for 5 days, to once-a-week over a number of weeks. The most common dosage was once-a-week sessions, with sessions lasting 1-2 h; however, timeframes ranged from 6-12 weeks [12,43-45]. Additionally, the studies by Heitzman-Powell et al [50] and Wacker et al [47] did not have finite periods, and the duration of intervention continued until all training modules were completed at the participants’ own pace. The lack of comparison regarding the dosage of education and training provided to the parents prevented the identification of an optimal amount of education and training to achieve the maximum benefit to the children.

The methods of delivery for the parent-mediated interventions were equally as wide-ranging, with Hamad et al [49], Heitzman-Powell et al [50], and the study by Ingersoll and Berger [43], Ingersoll et al [44], and Pickard et al [45], requiring parents to access resources on the Web and progress through the content at their own pace. Heitzman-Powell et al [50] coupled the Web-based modules with distant coaching sessions delivered by qualified clinicians. The studies by St. Peter et al [46], Wacker et al [47], and Vismara et al [48] utilized a telehealth delivery model with live, 2-way videoconferencing by qualified clinicians who delivered the intervention in isolation, coupled with a Web-based self-guided website or using teaching materials contained on a DVD. St. Peter et al [46] compared the difference between the effectiveness of delivery methods; training provided using video methods versus training provided via a written manual. Finally, the study by Ingersoll and Berger [43], Ingersoll et al [44], and Pickard et al [45] compared 2 groups; one receiving access to a Web-based training program only and the other having access to the same Web-based training program, but with additional weekly therapist-assistance via videoconferencing.

Identifying the superior delivery method of intervention for this population is limited by a lack of between-group comparisons within the included studies. Only the studies by Ingersoll and Berger [43], Ingersoll et al [44,] and Pickard et al [45], and St. Peter et al [46] had comparison groups. Methods with increased user interaction demonstrated some superiority with DVDs having higher adherence to the training program compared with written content. Furthermore, regular therapist-assisted sessions resulted in increased intervention completion, parent appropriateness of intervention, and improvements in parent knowledge and skills.

Overall, these findings suggest that training delivered to parents who live outside of urban areas or with limited access to services can have some effect in improving the social behavior and communication skills in their child with ASD and a large effect on increasing their own knowledge and skills in of ASD interventions. Additionally, no specific content or dosage can be identified as being superior; however, more interactive methods of delivery, such as videos and regular therapist contact for training have been proven to (1) improve adherence, (2) increase completion rates, and (3) improve fidelity in parent-mediated interventions.

### Risk of Bias in Included Studies

The St. Peter et al [46], Ingersoll and Berger [43], Ingersoll et al [44], and Pickard et al [45] studies assigned participants to different intervention groups. The remaining 5 articles have a high risk of selection bias. In the study by St. Peter et al [46], the randomisation process was poorly described with no mention of blinding and allocation procedures by the researchers. The authors reported homogeneity between samples with no significant differences in socioeconomic status, educational level, or previous experience with the intervention between the experimental and control groups and autism severity scores. Therefore, the risk of bias from confounding variables was reduced due to the homogeneity of the 2 groups. Confounding bias was addressed in the study by Ingersoll and Berger [43], Ingersoll et al [44], and Pickard et al [45] by matching participants on their pretreatment expressive language age using a standardized assessment prior to randomisation.

All 7 studies were subject to a high risk of bias due to a lack of blinding. Five of the studies in this review were at a higher risk of confounding bias due to the lack of controls. The small sample sizes of these articles increased the likelihood of type II errors with no article reporting a power calculation relative to the outcome measures.

## Discussion

### Principal Findings

Findings of this systematic review provide preliminary evidence that parent-mediated intervention training for families living in nonurban areas can assist in improving social behavior and communication skills of children with ASD. Weak study design, lack of standardized outcome measures, lack of measurement outcomes in children with ASD, small participant numbers, high risk of bias, and large variations in interventions limit the generalizability and conclusiveness of the findings to the target population. Despite the limitations, preliminary findings from this review suggest that parent-mediated intervention training delivered remotely could benefit both parents and children with ASD given the barriers they face in accessing traditional services.

The notion that parent-mediated interventions can fully address the gap of limited access to services and be an effective alternative intervention for children with ASD needs further investigation. A systematic review conducted by McConachie and Diggle [23] focused on parent-delivered interventions regardless of geographical location or method of delivery for children with ASD. The authors concluded that whereas these types of interventions can improve the social behavior and communication challenges of children with ASD, the lack of studies with robust study design limits the ability to draw further conclusions and highlighted the need for further research. This paper builds on these findings by reviewing current literature on the effectiveness of parent-mediated intervention training delivered remotely to a nonurban population who face a number of barriers accessing traditional services.

In this review, effect sizes were larger for intervention outcomes that targeted parents’ knowledge and intervention fidelity skills, compared with intervention outcomes to improve social behavior and communication skills for their children. Only 2 studies included measures of social behavior and communication skills in the children with ASD despite all the interventions providing training for parents to deliver therapy to address these skills. This finding indicates that parents have the potential to improve their knowledge and intervention fidelity skills and be agents in the delivery of therapeutic interventions, thereby improving the social behavior and communication skills of their children with ASD.

The results of this review indicate that the use of telehealth, Web-based modules, and DVDs all seem to have some effect in educating parents about ASD and increasing the fidelity in the delivery of interventions. A lack of standardized measurements and RCTs limited the comparison of interventions within this review. Interventions that were delivered using videos were more effective and accepted by parents than written information. Additionally, weekly contact with a therapist to answer questions and provide coaching proved to be more effective in the areas of (1) intervention appropriateness, (2) program completion, (3) parent intervention fidelity, (4) parent engagement, and (5) parent’s positive perception of their child, when compared to a self-directed program alone. Considering this, the interventions created for families that have limited access to face-to-face therapy could be tailored to meet the needs of the individual parents based on their proximity to services, personal qualities, resources, and preference. Furthermore, interventions clearly benefitted from regular contact with trained professionals throughout the training program.

Defining populations based on their geographical location is challenging due to differing methodologies and definitions adopted by different countries. This disparity in terminology and classification systems makes trying to understand the unique characteristics of families having a child with ASD and living in regional and remote areas difficult due to the wide variability of proximity and access to appropriate services. This is confounded when trying to compare populations from different countries that use vastly different classification systems. The review highlights the importance for researchers to use the relevant geographical classification system in their country to make defining study populations more clearly thereby providing better context for their study.

Finally, evidence is emerging that suggests there is indeed a significant difference in the characteristics and needs of families having a child with ASD residing in urban areas and those residing in rural areas, but further investigation is needed [16-18,22]. Intuitively this discrepancy between the populations makes sense; however, the poor description of participant characteristics, lack of control groups, and absence of comparisons between these 2 groups prevent conclusive findings.

### Recommendations for Future Research

Further research into the feasibility, efficacy, and appropriateness of the methods of delivery for this unique population will help inform clinical decisions. This systematic review provides preliminary evidence on the effectiveness of remotely delivered parent-mediated intervention training. However, more research is needed to determine the most effective balance between parent-mediated intervention and therapist support via Web-based or distance training to provide the best outcome for a child with ASD, while considering the family’s proximity to traditional services. Furthermore, investigation into the effectiveness of the parent-mediated intervention training should not only measure parents’ knowledge and skill attainment but also the intervention effectiveness in improving social behavior and communication skills of children with ASD.

Future experimental studies on the effectiveness of parent-mediated interventions, including training programs, should include (1) larger sample sizes, (2) RCTs, (3) improved controls for bias, and (4) use of standardized outcome measures. A lack of comparison groups prevented a meta-analysis in this review. Standardized outcome measures should be employed wherever possible, as these were seldom used in the included studies in this review, with nonvalidated measures often created by the researchers to evaluate the effectiveness of their own intervention. This increased the risk of bias in the studies, thus limiting the impact of the studies’ findings. Further research could be focused on comparing different parent training interventions, their components, dosage, and the methods of delivery to determine a superior strategy in increasing parent knowledge and intervention fidelity while improving social behavior and communication skills of their children with ASD.

Despite the studies reporting on the parents’ perceived appropriateness and overall satisfaction of the intervention, there was limited investigation into the influences of parent engagement in the parent-mediated interventions. Further research in relation to the factors surrounding parent engagement in the intervention could help inform clinicians when devising training interventions related to content, parent commitment, and methods of delivery.

There is emerging evidence that interventions delivered remotely can improve the socioemotional and communication skills of children with ASD and may be an alternative to traditional models of therapy [11,56]. The appropriateness and feasibility for parents to utilize other methods to deliver therapy to their children such as direct one-on-one interventions using telehealth technology or the ever-expanding suite of tablet and other ICT-based interventions remains to be comprehensively investigated. Finally, economic modeling comparing the expense of a variety of methods of delivery and interventions could help inform the most cost-effective and feasible delivery method.

The unique context in which families having children with ASD and living in nonurban settings needs to be further researched. Emerging evidence suggests that the nonurban context is different, yet, the unique enablers and barriers in relation to service delivery that these families experience are yet to be fully understood. Furthermore, there is a need for comparison studies between urban and nonurban populations to better develop effective, appropriate, and feasible interventions to improve the social behavior and communication skills in children with ASD; thus allowing the development of tailor-made interventions for each population.

### Limitations

A rigorous process involving (1) the searching of 5 databases, (2) establishing interrater reliability between 2 independent researchers for inclusion or exclusion agreements, (3) standardized data extraction forms, and (4) methodological assessment using the Kmet appraisal checklist was conducted in this study. Despite this, there are some limitations in the review that should be noted. Defining the population was challenging given the poor use of standardized geographical classification systems by authors. Inclusion was based on author report of the participants living in areas described as nonurban, rural, or remote, and as having limited access to services. This could have led to some studies being excluded if this description was not provided by the authors. Additionally, the small number of articles included limits the generalizability of findings to the target population.

### Conclusions

Overall, there is preliminary evidence that parent-mediated intervention training delivered remotely can improve parents’ knowledge in ASD, parent intervention fidelity, and subsequently improve the social behavior and communication skills of their children with ASD. The studies included in this review had an unclear or high risk of bias due to a lack of control groups and paucity of using standardized outcome measures. Additionally, difficulties in defining the participant characteristics limited the translatability to the target population. Few studies reported on the feasibility and appropriateness of the interventions and the factors of parent engagement in the interventions were evident in most studies. Future research should aim to use RCT designs, incorporate standardized outcome measures, and describe participant characteristics in greater detail. Furthermore, the review highlighted the need to investigate the feasibility and appropriateness of the interventions in addition to the factors influencing parent engagement in the interventions.

## Abbreviations

ASD: Autism spectrum disorder
DSM-V: diagnostic and statistical manual of mental disorders
ICT: information communication technology
RCT: randomized controlled trial
